# Modified Ewald Summation Method for Slab Geometries: Application to Donnan Potentials, Confinement Electrostatics, and Titration Simulations

**DOI:** 10.1021/acs.jpcb.6c05163

**Published:** 2026-09-23

**Authors:** Yan Levin, Amin Bakhshandeh

**Affiliations:** † Instituto de Física, Universidade Federal do Rio Grande do Sul, Caixa Postal 15051, Porto Alegre, Rio Grande do Sul CEP 91501-970, Brazil; ‡ Escola de Química e Alimentos, Universidade Federal do Rio Grande, Rio Grande 96203-900, Brazil

## Abstract

Simulating electrochemical systems confined within nanoscale slab geometries is a major challenge due to long-range electrostatic interactions and the breakdown of global charge neutrality when the domain exchanges ions with an external bulk electrolyte reservoir. In this work, we present a grand-canonical simulation framework designed for slab geometries that utilizes a regularized 3D periodic Ewald summation technique to systematically converge to the true 2D periodic Green’s function without unphysical cell-width (*L*_*z*_) dependencies. Unlike traditional grand canonical methods that rely on neutral-pair insertions to maintain a zero net charge, our framework allows the net cell charge to fluctuate by implementing decoupled, single-ion trial moves. This approach bypasses the cooperative sampling bottlenecks that plague neutral-pair insertions inside dense, strongly charged nanopores. Crucially, the method allows us to calculate the Donnan potential (φ_D_) inside the cell, which cannot be accessed directly by any other available simulation method. The Donnan potential acts as a direct microscopic analog to the electromotive force (emf) of a nanoscale electrochemical cell, making the method uniquely suited for iontronic and nanofluidic applications where nonideal ion–ion correlations and strong inhomogeneities modify device potentials. The methodology is validated against exact solutions of the nonlinear Poisson–Boltzmann equation, demonstrating excellent agreement for both local ionic density profiles and self-consistent potentials. Finally, we demonstrate the utility of this framework by simulating charge regulation and surface titration of weak acidic groups at the pore wall.

## Graphical abstract

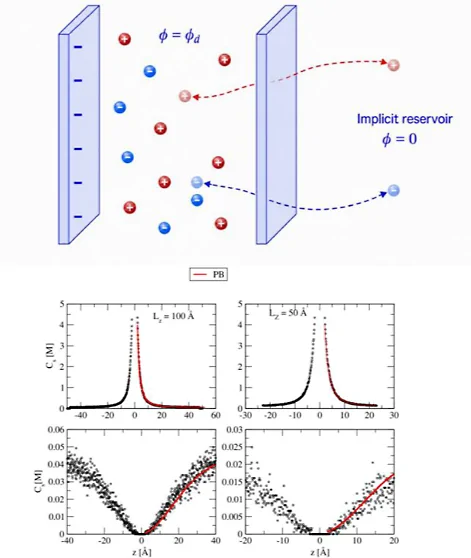

## 1 Introduction

The physical chemistry of fluids confined within nanoscale slab geometries governs a vast array of processes, spanning heterogeneous catalysis, energy storage materials, and cellular biophysics.^1–7^ In chemical applications, slab models serve as a foundational framework for studying thin films, solid–liquid surfaces, and interfacial properties.^8–10^ For instance, in modeling catalyst surfaces, a standard convention keeps the bottom atomic layers fixed to mimic the bulk material substrate while allowing the top layers and chemical adsorbates to relax dynamically.^11–15^ Similarly, in electrochemical energy devices such as high-performance batteries, complex porous electrode architectures create confined planar geometries that directly dictate ion transport, double-layer capacitance, and overall charge storage capabilities.^16–19^

Beyond material science, long-range electrostatic interactions under spatial confinement dictate the behavior of soft matter and living biological systems.^20–27,27–34^ Cellular viability relies inherently on semipermeable membranes that maintain strict electrochemical gradients by regulating specific pathways based on ionic selectivity. Such selective transport boundaries are not only fundamental to biological compartmentalization but also drive widespread industrial engineering applications, including nanofiltration and desalination for water treatment.^35–38^ In both natural and synthetic systems,^39–43^ the restriction of mobile ionic species across a boundary generates an interfacial electrostatic potential drop. This phenomenon was first treated thermodynamically by Donnan in 1911 within the classical framework of macroscopic equilibrium phase behavior.^25^ More recently, manipulating and measuring this nanoscale potential drop has become of great importance for nanofluidics and iontronics, where confined electrolyte channels gate functional ionic currents, and the resulting macroscopic Donnan potential acts as a direct microscopic analog to the open-circuit electromotive force (emf) of an electrochemical cell.^44–48^

Developing a molecular, microscopic description of these interfacial regions is notoriously difficult due to the long-range nature of Coulombic interactions. Standard theoretical benchmarks, such as Derjacuin–Landau–Verwey–Overbeek (DLVO) theory and the classical Poisson–Boltzmann equation, provide critical insights but are fundamentally bound to the mean-field level.^49–53^ They omit local ion–ion correlations, discrete solvent packing, and fluctuating polarization fields—effects that aggressively reshape local electric fields under strong nanoscale confinement. Explicit molecular simulations are therefore essential to capture these microstructural correlations.

For bulk systems, long-range forces are successfully managed using the standard 3D periodic Ewald summation technique,^54–59^ which splits the Coulomb potential into a rapidly decaying real-space sum and a smooth reciprocal-space sum over periodic replica lattice. An obstacle, however, emerges, when simulating confined slab systems in thermodynamic equilibrium with an external electrolyte reservoir.^60^ In an open ensemble, see Figure 1, such as a slit nanopore or a semipermeable membrane channel, balancing chemical potentials requires a responsive influx or efflux of charge. When a local net charge fluctuation occurs or when an applied chemical potential difference maintains an asymmetric distribution of ions across a boundary, the physical domain inside the slit pore becomes locally non-neutral. Because the conventional 3D Ewald method is mathematically derived for a periodic cell enveloped by a uniform neutralizing background, its direct application to such open geometries introduces severe physical artifacts. Specifically, the unphysical neutralizing background charge density (ρ_back_ = −*Q*_net_/*V*) fills the entire simulation box uniformly. In a slab geometry bounded by a wide vacuum gap along the confinement axis, this means the empty vacuum regions are artificially loaded with a net background charge. When periodically replicated, these charged vacuum spaces form unphysical sheets of background charge separating the periodic images of the physical slabs affecting the local electrostatic potential.

**Figure 1:**
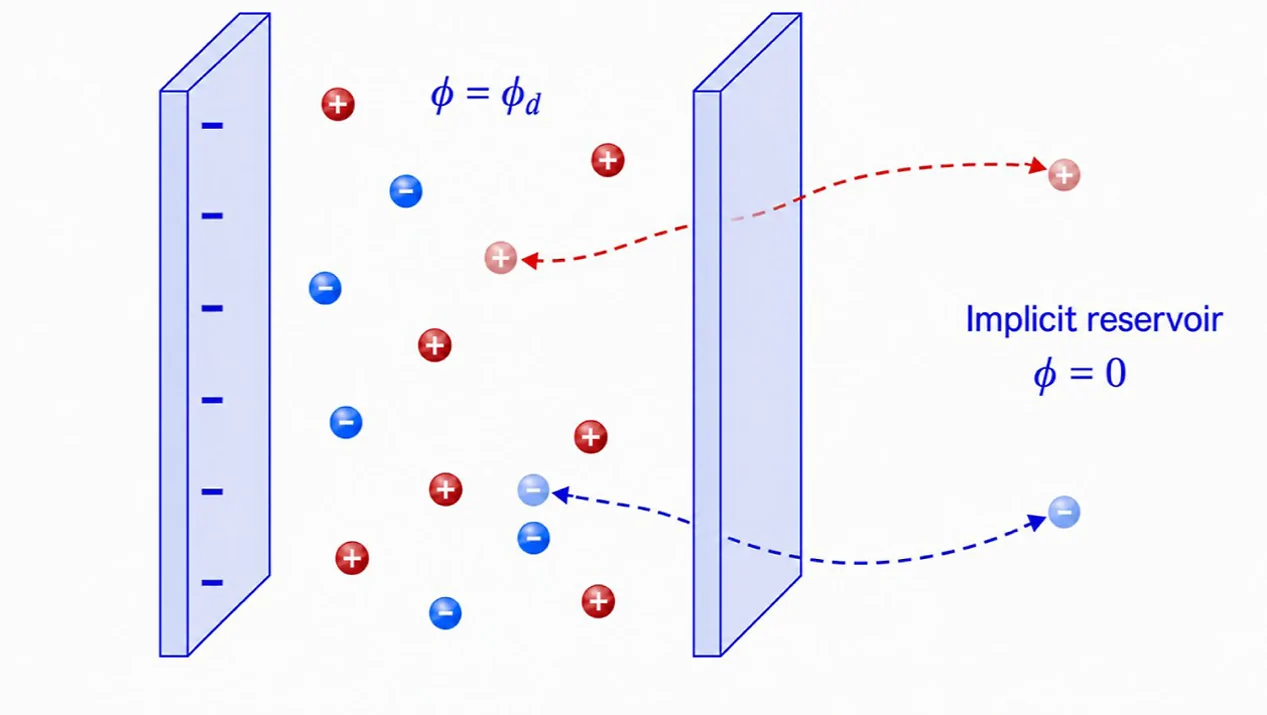
Schematic showing a slab-like system in contact with an external reservoir. Note that the electrostatic potential inside the reservoir is 0 while the dynamics of ionic diffusion results in a Donnan potential difference across the system-reservoir interface.

To rectify these periodic artifacts while preserving the computational efficiency of Ewald sums, various techniques have emerged. One alternative is to employ explicit 2D periodic electrostatics, such as Lekner summation methods or direct 2D Ewald summation variants.^61–63^ While mathematically elegant, these explicit 2D methods suffer from severe computational bottlenecks because their real- and reciprocal-space terms involve slowly converging series or complex transcendental functions that do not scale efficiently for large systems. Consequently, explicit 2D periodic sums are significantly slower than modern 3D Ewald implementations, which benefit from decades of algorithmic optimization, such as particle-Mesh Ewald (PME) techniques.^64–67^ To bypass the speed limitations of 2D methods, a common alternative is to utilize a fast 3D Ewald engine combined with a wide vacuum gap along the confinement axis and an electrostatic correction term, such as the Yeh–Berkowitz method.^54^ While highly effective for closed, canonical slab geometries with a fixed net charge,^68,69^ these approaches encounter major consistency challenges when applied in a grand-canonical setting with individual ion moves.

Grand canonical Monte Carlo (GCMC) implementations traditionally rely on strict neutral-pair insertion and deletion steps to preserve an absolute zero net charge at every single simulation configuration.^60^ Inside highly confined domains or dense, intensely charged double layers, these coupled trials suffer from a severe cooperative bottleneck; the probability of simultaneously finding hard-sphere voids for both an anion and a cation drops precipitously, stalling phase-space sampling with vanishingly low acceptance rates. Neutral-pair moves also introduce severe physical distortions within strong screening clouds, forcing co-ions into energetically unfavorable regions of intense repulsion simply to balance a local counterion insertion.

Rather than relying on computationally slow Lekner-like 2D summations or structurally restrictive neutral-pair exchanges, the methodology we develop in this work introduces a regularized periodic Green function approach that modifies the highly efficient 3D Ewald summation to account rigorously for an open slab geometry in thermodynamic equilibrium with a reservoir, Figure 1. The single-ion insertion and deletion moves allow the net charge inside the simulation cell, *Q*_t_, to fluctuate naturally bypassing the pair-insertion bottleneck and forcing ions to correctly respond to local electric fields. By incorporating both the structural Bethe potential arising from 3D periodicity of Ewald summation and explicit Donnan boundary conditions, our self-consistent feedback algorithm dynamically tunes the macroscopic Donnan potential (φ_D_) to drive the system toward global neutrality *on average*. This approach yields an advantage for iontronic modeling: φ_D_ is extracted directly as a primary thermodynamic parameter, providing a direct, highly accurate route to measure the nonideal electrostatic correlation corrections that reshape a cell’s emf under extreme nanoscale confinement. As the cell width (*L*_*z*_) increases, the unphysical 3D periodic background artifacts disappear, systematically converging to the true 2D periodic limit. We validate the mathematical framework by comparing ionic density profiles and self-consistent Donnan potentials directly against exact solutions of the nonlinear Poisson–Boltzmann equation for 1:1 electrolyte, demonstrating excellent agreement. Crucially, this electrostatics foundation is structurally general: it provides the necessary mathematical framework to perform Grand Canonical and reactive Monte Carlo simulations of responsive interfaces, such as weak acidic groups functionalized at the pore walls (±*L*_w_/2), where local titration states fluctuate self-consistently with discrete single-charge configurations.

The remainder of this paper is organized as follows. In Section 2, we detail the Green function derivation and the regularization of the 3D background terms. Section 3 outlines the Grand Canonical and reactive titration simulation framework. Section 4 presents the simulation model, numerical validation against Poisson–Boltzmann theory, and a thorough discussion of the background-induced parallel-plate capacitor artifacts that plague uncorrected implementations. Section 5 establishes the single-plate responsive surface titration model, outlines the sampling and iontronic application advantages of independent single-ion moves, and highlights the validation of our framework against our previously developed analytical theory under monovalent electrolyte conditions. Finally, concluding remarks and future outlooks are presented in Section 6.

## 2 Electrostatic Framework and Green Function Derivation

### 2.1 Two-Dimensional Periodic Reference System

We begin by establishing the electrostatic potential generated by a discrete source charge in a two-dimensional (2D) periodic slab geometry. Consider a source ion of charge *q*_*i*_ located at position **r**_*i*_ = (*x*_*i*_, *y*_*i*_, *z*_*i*_) within a rectangular simulation cell that is periodically replicated infinitely along the *x* and *y* directions with periodicities *L*_*x*_ and *L*_*y*_, respectively. To satisfy global electrostatics within this reference plane, the ion and its replicas are accompanied by a uniform neutralizing background surface charge density σ_back_ = −*q*_*i*_/(*L*_*x*_ *L*_*y*_).

The electrostatic potential at an arbitrary observation point **r** = (*x*, *y*, *z*) satisfies the 3D Poisson equation under 2D periodic boundary conditions which in Gaussian units takes the form

1 $$\nabla^{2}G_{2d}\left(r,r_{i}\right)=-\frac{4\pi q_{i}}{\epsilon_{w}}\left[\sum_{m_{x},m_{y}=-\infty }^{\infty }\delta \left(r-r_{i}+m_{x}L_{x}\mathrm{x̂}+m_{y}L_{y}ŷ\right)-\frac{\delta \left(z-z_{i}\right)}{L_{x}L_{y}}\right]$$

where ϵ_w_ is the dielectric constant of the background medium. By expanding the lateral coordinates into a 2D Fourier series, the exact solution to eq 1 is expressed as

2 $$G_{2d}\left(r,r_{i}\right)=\frac{1}{L_{x}L_{y}}\sum_{m\neq 0}g_{m}\left(z_{i},z\right)\cos\left[\frac{2\pi m_{x}}{L_{x}}\left(x-x_{i}\right)+\frac{2\pi m_{y}}{L_{y}}\left(y-y_{i}\right)\right]$$

where the 1D transverse Fourier component *g*_**m**_(*z*_*i*_, *z*) along the confinement axis is

3 $$g_{m}\left(z_{i},z\right)=\frac{2\pi q_{i}e^{-k\left|z-z_{i}\right|}}{\epsilon_{w}k}$$

The horizontal wavevector magnitude is defined by $k=2\pi \sqrt{m_{x}^{2}/L_{x}^{2}+m_{y}^{2}/L_{y}^{2}}$ , and the notation **m** ≠ **0** explicitly excludes the *k* = 0 mode. As seen directly from the structure of eq 1, the uniform background charge density is responsible for identically canceling this singular *m* = 0 term.

To make this framework physically applicable to non-neutral systems, we introduce a regularized 2D Green’s function, *G*_2dr_, which isolates the potential of the explicit discrete ions. This is achieved by subtracting the continuous potential contribution generated by the uniform background sheet. Because an infinite, delocalized planar charge distribution introduces a divergent potential contribution, this background subtraction effectively regularizes the Green’s function by eliminating a macroscopic electrostatic infinity. Performing this background subtraction yields

4 $$G_{2dr}\left(r,r_{i}\right)=\frac{1}{L_{x}L_{y}}\sum_{m\neq 0}g_{m}\left(z_{i},z\right)\cos\left[\frac{2\pi m_{x}}{L_{x}}\left(x-x_{i}\right)+\frac{2\pi m_{y}}{L_{y}}\left(y-y_{i}\right)\right]-\frac{2\pi q_{i}}{\epsilon_{w}L_{x}L_{y}}\left|z-z_{i}\right|$$

The real-space expression for the 2D background subtraction potential, $-\frac{2\pi q_{i}}{\epsilon_{w}L_{x}L_{y}}\left|z-z_{i}\right|$ , stems from integrating the 1D Poisson equation along the confinement axis for an infinite planar sheet of charge density σ_back_.

### 2.2 Mapping onto a Regularized 3D Ewald Summation

While eq 4 provides a mathematically rigorous expression for the 2D periodic potential, it suffers from severe real-space convergence bottlenecks when the source and observation coordinates share identical or highly similar positions along the *z*-axis (*z* ≈ *z*_*i*_). This poor real-space convergence renders direct evaluations numerically inefficient for molecular simulations. Conversely, 3D periodic electrostatics can be evaluated with exceptional efficiency. Our strategy is therefore to map the 2D periodic slab system onto a 3D periodic lattice by introducing an artificial periodicity *L*_*z*_ along the confinement axis separated by a sufficiently large vacuum gap (*L*_*z*_ ≫ *L*_*x*_, *L*_*y*_).

For an ion periodically replicated in all three dimensions within a uniform neutralizing 3D background charge density ρ_q_ = *q*_*i*_/*V* (where *V* = *L*_*x*_ *L*_*y*_ *L*_*z*_), the standard 3D Ewald summation splits the electrostatic potential into short-range real-space and long-range reciprocal-space sums

5 $$G_{3d}\left(r,r_{i}\right)=q_{i}\sum_{n}\frac{erfc\left(\kappa_{e}\left|r-r_{i}-n\cdot L\right|\right)}{\epsilon_{w}\left|r-r_{i}-n\cdot L\right|}+\frac{4\pi q_{i}}{V}\sum_{k}\frac{e^{-k^{2}/4\kappa_{e}^{2}}}{\epsilon_{w}k^{2}}e^{ik\cdot \left(r-r_{i}\right)}+\frac{1}{V}\sum_{k}{\mathrm{\phi ̃}}_{b}\left(k\right)e^{ik\cdot \left(r-r_{i}\right)}$$

where κ_e_ is the Ewald screening parameter, **n** = (*n*_*x*_, *n*_*y*_, *n*_*z*_) represents the 3D lattice vectors and **L** = (*L*_*x*_, *L*_*y*_, *L*_*z*_). The reciprocal-space wavevectors are defined as **k** = (*k*_*x*_, *k*_*y*_, *k*_*z*_) = (2π*m*_*x*_/*L*_*x*_, 2π*m*_*y*_/*L*_*y*_, 2π*m*_*z*_/*L*_*z*_), where **m** = (*m*_*x*_, *m*_*y*_, *m*_*z*_) is a vector of integers, and its magnitude is denoted by *k* = |**k**|. The third term represents the potential generated by the uniform neutralizing background block centered on the ion’s position

6 $${\mathrm{\phi ̃}}_{b}\left(k\right)=-\frac{4\pi q_{i}}{V\epsilon_{w}}\frac{\int_{V}e^{-ik\cdot \left(r-r_{i}\right)}dr}{k^{2}}$$

The **k** = **0** term of the reciprocal sum in eq 5 is singular and must be treated by a rigorous limiting procedure.^70^ Evaluating this limit under standard spherical boundary conditions yields

7 $$G_{3d}\left(r,r_{i}\right)=q_{i}\sum_{n}\frac{erfc\left(\kappa_{e}\left|r-r_{i}-n\cdot L\right|\right)}{\epsilon_{w}\left|r-r_{i}-n\cdot L\right|}+\frac{4\pi q_{i}}{V}\sum_{k\neq 0}\frac{e^{-k^{2}/4\kappa_{e}^{2}}}{\epsilon_{w}k^{2}}e^{ik\cdot \left(r-r_{i}\right)}-\frac{\pi q_{i}}{\epsilon_{w}V\kappa_{e}^{2}}+\phi_{B}$$

where ϕ_B_ represents the structural Bethe potential encoding the shape constraints of the macroscopic crystalline boundaries.^70^ It arises from a nonzero quadrupole moment inside the periodic cell, which leads to a dipole layer at the macroscopic boundary of the Ewald crystal.

For slab geometries, we require a planewise summation layout, where periodic charges along the lateral *x* and *y* dimensions are summed completely before extending the boundaries along the transverse *z*-axis. Because standard 3D Ewald summation enforces a spherical macro-boundary via its structural singular limit, it is impossible to alter the summation geometry directly within the standard reciprocal sum. To resolve this structural mismatch, we explicitly evaluate the Bethe potential ϕ_B_ for a slab supercell configuration. We consider a supercell lattice composed of an infinite 2D plane of periodic charges centered at *z* = 0, bounded by a 3D background cloud spanning from *z* = −*L*_*z*_/2 to *z* = *L*_*z*_/2, and periodically replicated along the *z*-axis. At macroscopic distances, the discrete planar charges compress into an effective uniform surface charge density σ = *q*_*i*_/(*L*_*x*_ *L*_*y*_). Calculating the average electrostatic potential inside the supercell yields the Bethe potential for a periodic crystal composed of replicated slabs

8 $$\phi_{B}=\frac{1}{L_{x}L_{y}L_{z}}\int_{-L_{x}/2}^{L_{x}/2}dx\int_{-L_{y}/2}^{L_{y}/2}dy\int_{-L_{z}/2}^{L_{z}/2}dz\left(\frac{2\pi q_{i}}{\epsilon_{w}L_{x}L_{y}L_{z}}z^{2}-\frac{2\pi q_{i}}{\epsilon_{w}L_{x}L_{y}}\left|z\right|\right)=-\frac{q_{i}\pi L_{z}}{3\epsilon_{w}L_{x}L_{y}}$$

where the first quadratic term in the integrand isolates the potential of the uniform 3D background, and the second linear term isolates the potential of the central planar sheet.

We can now construct an intermediate regularized 3D Ewald Green’s function, $G_{3dr}^{\mathrm{int}}$ , by explicitly subtracting the parabolic potential artifact produced by the uniform 3D background charge density centered on the source coordinate. Mirroring our 2D formulation, this subtraction removes the divergent potential of an infinite charge distribution, effectively regularizing the Green’s function by eliminating a macroscopic electrostatic infinity from the system. This yields

9 $$G_{3dr}^{\mathrm{int}}\left(r,r_{i}\right)=q_{i}\sum_{n}\frac{erfc\left(\kappa_{e}\left|r-r_{i}-n\cdot L\right|\right)}{\epsilon_{w}\left|r-r_{i}-n\cdot L\right|}+\frac{4\pi q_{i}}{V}\sum_{k\neq 0}\frac{e^{-k^{2}/4\kappa_{e}^{2}}}{\epsilon_{w}k^{2}}e^{ik\cdot \left(r-r_{i}\right)}-\frac{\pi q_{i}}{\epsilon_{w}V\kappa_{e}^{2}}-\frac{2\pi q_{i}}{\epsilon_{w}V}{\left(z-z_{i}\right)}^{2}+\phi_{B}$$

An alternative regularization procedure for the electrostatic energy has been provided in ref 69.

### 2.3 Madelung Constant Validation

To verify the mathematical accuracy of replacing the exact 2D periodic Green’s function with our regularized 3D Ewald formulation under wide vacuum conditions, we calculate the electrostatic energy per particle of a square one-component 2D plasma lattice. The exact analytic Madelung energy for this reference geometry is known precisely to be

10 $$u=-\frac{Mq^{2}}{2L\epsilon_{w}}$$

where *M* = 3.9002649200019555...is the exact 2D Madelung constant, and *L* is the lateral lattice spacing (*L*_*x*_ = *L*_*y*_ = *L*). Utilizing our regularized 3D formulation, the numerical approximation is evaluated via the self-potential limit

11 $$u_{approx}=\frac{q}{2}\lim_{r\to r_{i}}\left(G_{3dr}^{\mathrm{int}}\left(r,r_{i}\right)-\frac{q}{\epsilon_{w}\left|r-r_{i}\right|}\right)\equiv -\frac{M_{approx}q^{2}}{2L\epsilon_{w}}$$

Expanding this limit explicitly into its real and reciprocal Ewald components leads to

12 $$u_{approx}=\frac{2\pi q^{2}}{L^{2}L_{z}\epsilon_{w}}\sum_{n\neq 0}\frac{\exp\left[-\frac{4\pi^{2}}{4\kappa_{e}^{2}}\left(\frac{n_{x}^{2}}{L^{2}}+\frac{n_{y}^{2}}{L^{2}}+\frac{n_{z}^{2}}{L_{z}^{2}}\right)\right]}{4\pi^{2}\left(\frac{n^{2}}{L^{2}}+\frac{m^{2}}{L^{2}}+\frac{p^{2}}{L_{z}^{2}}\right)}-\frac{q^{2}\kappa_{e}}{\epsilon_{w}\sqrt{\pi }}+\frac{q^{2}}{2\epsilon_{w}}\sum_{n\neq 0}\frac{erfc\left(\kappa_{e}\sqrt{n_{x}^{2}L^{2}+n_{y}^{2}L^{2}+n_{z}^{2}L_{z}^{2}}\right)}{\sqrt{n_{x}^{2}L^{2}+n_{y}^{2}L^{2}+n_{z}^{2}L_{z}^{2}}}-\frac{\pi q^{2}}{2L^{2}L_{z}\epsilon_{w}\kappa_{e}^{2}}-\frac{q^{2}\pi L_{z}}{6\epsilon_{w}L^{2}}$$

Evaluating eq 12 across varying cell aspect ratios yields the following results for the computed Madelung constant:

- For *L*/*L*_*z*_ = 1: *M*_approx_ = 3.88449503068
- For *L*/*L*_*z*_ = 1/2: *M*_approx_ = 3.90023691285
- For *L*/*L*_*z*_ = 1/3: *M*_approx_ = 3.90026486804

This systematic convergence demonstrates that our regularized 3D Green’s function matches the exact 2D periodic limit with high precision as the artificial periodicity along the *z*-direction increases relative to the lateral dimensions.

### 2.4 Reservoir Boundary Condition Matching

Equation 4 defines a valid 2D periodic potential, however, to simulate Donnan equilibrium, the confined slab must be placed in direct contact with a macroscopic electrolyte reservoir whose electrostatic potential is defined as zero (ϕ_res_ = 0). To proceed we place the slab system to occupy the region −*L*_w_/2 < *z* < *L*_w_/2, with the reservoir connected to the system at the interface located at *z* = +*L*_w_/2, see Figure 1.

To satisfy equilibrium with this bulk reference phase, the planar-averaged electrostatic potential must vanish identically at the contact interface (*z* = +*L*_w_/2). Taking the spatial average of the regularized 2D function over the lateral coordinates (*x*, *y*), the periodic cosine modes vanish, leaving only the 1D background subtraction term

13 $${\left\langle G_{2dr}\left(\frac{L_{w}}{2},z_{i}\right)\right\rangle }_{x,y}=-\frac{2\pi q_{i}}{\epsilon_{w}L_{x}L_{y}}\left(\frac{L_{w}}{2}-z_{i}\right)$$

where ⟨···⟩_*x*,*y*_ denotes the spatial average over the lateral area, and we have noted that the source coordinate satisfies *z*_*i*_ ≤ *L*_w_/2. Therefore, $G_{3dr}$ does not satisfy the required boundary condition. We note, however, that eq 13 corresponds directly to a 1D Green function associated with the Laplace operator. We can then implement the correct boundary conditions without altering the internal discrete source terms by adding a general solution to the homogeneous 1D Laplace equation whose second derivative with respect to *z* vanishes. To impose the reservoir boundary condition ${\left\langle G_{2dr}\left(+L_{w}/2,z_{i}\right)\right\rangle }_{x,y}=0$ self-consistently for all internal source positions *z*_*i*_, we introduce the linear correction

14 $$\psi_{corr}\left(z,z_{i}\right)=-\frac{2\pi q_{i}}{\epsilon_{w}L_{x}L_{y}}\left(z+z_{i}\right)+\frac{2\pi q_{i}L_{w}}{\epsilon_{w}L_{x}L_{y}}$$

By incorporating this boundary matching constraint alongside the geometric Bethe potential [eq 8], the final regularized Green function $G_{3dr}$ tailored for Donnan simulations is established as

15 $$G_{3dr}\left(r,r_{i}\right)=q_{i}\sum_{n}\frac{erfc\left(\kappa_{e}\left|r-r_{i}-n\cdot L\right|\right)}{\epsilon_{w}\left|r-r_{i}-n\cdot L\right|}+\frac{4\pi q_{i}}{V}\sum_{k\neq 0}\frac{e^{-k^{2}/4\kappa_{e}^{2}}}{\epsilon_{w}k^{2}}e^{ik\cdot \left(r-r_{i}\right)}-\frac{\pi q_{i}}{\epsilon_{w}V\kappa_{e}^{2}}-\frac{2\pi q_{i}}{\epsilon_{w}V}{\left(z-z_{i}\right)}^{2}-\frac{2\pi q_{i}}{\epsilon_{w}L_{x}L_{y}}\left(z+z_{i}\right)+\frac{2\pi q_{i}L_{w}}{\epsilon_{w}L_{x}L_{y}}-\frac{q_{i}\pi L_{z}}{3\epsilon_{w}L_{x}L_{y}}$$

### 2.5 Total Electrostatic Energy Functional

The total electrostatic energy *E* of a system containing *N* discrete particles evaluated via our reservoir-matched Green’s function is given by omitting the self-interaction terms (*i* = *j*) from the configuration double sum

16 $$E=\frac{1}{2}\sum_{i\neq j}^{N}q_{i}q_{j}G_{3dr}\left(r_{i},r_{j}\right)+\frac{1}{2}\sum_{i=1}^{N}q_{i}\lim_{r\to r_{i}}\left[G_{3dr}\left(r,r_{i}\right)-\frac{q_{i}}{\epsilon_{w}\left|r-r_{i}\right|}\right]$$

Expanding eq 16 explicitly into its core real-space, reciprocal-space, and structural charge components yields the finalized global energy functional

17 $$E=\sum_{k\neq 0}\frac{2\pi }{\epsilon_{w}V|k{|}^{2}}\exp\left[-\frac{|k{|}^{2}}{4\kappa_{e}^{2}}\right]\left[A{\left(k\right)}^{2}+B{\left(k\right)}^{2}\right]+\frac{1}{2}\sum_{i\neq j}^{N}\frac{q_{i}q_{j}erfc\left(\kappa_{e}\left|r_{i}-r_{j}\right|\right)}{\epsilon_{w}\left|r_{i}-r_{j}\right|}+\frac{2\pi }{\epsilon_{w}V}\left[M_{z}^{2}-Q_{t}G_{z}\right]-\frac{\kappa_{e}\sum_{i}q_{i}^{2}}{\epsilon_{w}\sqrt{\pi }}-\frac{\pi Q_{t}^{2}}{2\varepsilon_{w}V\kappa_{e}^{2}}-\frac{\pi Q_{t}^{2}L_{z}}{6\epsilon_{w}L_{x}L_{y}}+\frac{\pi Q_{t}^{2}L_{w}}{\epsilon_{w}L_{x}L_{y}}-\frac{2\pi Q_{t}M_{z}}{\epsilon_{w}L_{x}L_{y}}$$

where the collective wave structure factors and multipolar charge distribution moments are defined as

18 $$\begin{array}{lll} A\left(k\right) & = & \sum_{i=1}^{N}q_{i}\;\cos\left(k\cdot r_{i}\right)\\ B\left(k\right) & = & -\sum_{i=1}^{N}q_{i}\;\sin\left(k\cdot r_{i}\right)\\ Q_{t} & = & \sum_{i=1}^{N}q_{i}\\ M_{z} & = & \sum_{i=1}^{N}q_{i}z_{i}\\ G_{z} & = & \sum_{i=1}^{N}q_{i}z_{i}^{2} \end{array}$$

The structure of eq 17 offers key computational and physical insights. The first two terms represent the conventional 3D Ewald reciprocal- and real-space contributions, which can be calculated efficiently using standard mesh-based techniques such as particle-Mesh Ewald (PME). The remaining terms account explicitly for the global non-neutrality of the slab (*Q*_t_ ≠ 0), removing the unphysical background artifacts while self-consistently matching the electrostatic potential to zero at the reservoir interface (*z* = +*L*_w_/2). In the numerical implementation of the global energy functional *E*, we employ the standard minimum image convention for the short-range real-space interactions, which requires setting the Ewald screening parameter to κ_e_ ≥ 5/min­{*L*_*x*_, *L*_*y*_} to guarantee that the complementary error function decays to negligible values within the simulation cell. Crucially, this regularized energy functional remains valid for states with an arbitrary net system charge *Q*_t_, making it directly applicable for grand canonical and reactive simulation ensembles.

## 3 Grand Canonical and Reactive Titration Simulations

### 3.1 Ion Exchange and Donnan Acceptance Criteria

To simulate a confined electrochemical slab domain in a thermodynamic equilibrium with an external electrolyte reservoir containing salt and acid, we employ a grand canonical Monte Carlo (GCMC) framework. The simulation cell explicitly allows for individual ion insertion and deletion moves. Within the confined slit pore, fixed structural charges (such as macroions, colloids, or functionalized electrodes) are geometrically trapped and cannot cross the interfacial boundary to escape into the reservoir. Conversely, the small mobile electrolyte species are free to exchange with the bulk phase. Because the structural components cannot partition into the reservoir, a macroscopic electrostatic potential difference – the Donnan potential, φ_D_ – develops between the physical slab and the external bulk phase.

Crucially, because our regularized Green’s function $G_{3dr}$ is explicitly matched to an external bulk potential of zero at the contact interface (*z* = +*L*_w_/2), any mobile ion of charge *q*_*i*_ entering or leaving the slab through this boundary performs mechanical work against this potential drop, contributing an energy penalty or reward equal to *q*_*i*_φ_D_. Accounting for this interfacial work term, the grand canonical acceptance probabilities for ion insertion and deletion trials are given by min­(1, ϕ_add_) and min­(1, ϕ_rem_), respectively, with the transition parameters defined as^70,71^

19 $$\begin{array}{ll} \phi_{add} & =\frac{c^{⊖}V_{p}10^{-{pX}_{i}}}{N_{i}+1}\exp\left[-\beta \left(\Delta E_{ele}+q_{i}\varphi_{D}\right)\right]\\ \phi_{rem} & =\frac{N_{i}10^{{pX}_{i}}}{V_{p}c^{⊖}}\exp\left[-\beta \left(\Delta E_{ele}-q_{i}\varphi_{D}\right)\right] \end{array}$$

Here, Δ*E*_ele_ represents the change in global electrostatic energy computed via the regularized functional [eq 17] upon adding or removing an ion of type *i*, *N*_*i*_ is the instantaneous number of particles of that species inside the slit pore, *V*_p_ = *L*_*x*_ *L*_*y*_ *L*_w_ is the pore volume, β = 1/*k*_B_ *T*, and *c* ^⊖^ = 1 M is the standard reference state concentration. The chemical potential of species *i* in the external reservoir is defined conventionally as $\mu_{i}=\mu_{i}^{⊖}+k_{T}\;\ln\left(c_{i}/c^{⊖}\right)+\mu_{i}^{ex}$ , where *c*_*i*_ is the bulk reservoir concentration and μ_i_^ex^ is the excess chemical potential capturing nonideal interactions. To simplify the notation the reservoir chemical potential is introduced via the ion activity index pX_*i*_ ≡ – log_10_(*X*_*i*_/*c* ^⊖^), where the dimensionless bulk activity *X*_*i*_ is rigorously defined in terms of the local excess chemical potential as

20 $$X_{i}=c_{i}\;\exp\left(\beta \mu_{i}^{ex}\right)$$

To evaluate μ_i_^ex^ and establish the exact relation between the activity index pX_*i*_, the nominal reservoir concentrations, and the bulk pH, one may perform a separate grand canonical simulation of the bulk phase. In practice, however, we find that, for monovalent ions, the mean spherical approximation (MSA), coupled with the Carnahan–Starling (CS) description of the short-range excluded-volume interactions,^72–74^ provides an exceptionally accurate analytical expression for the excess chemical potential. Within this approximation, the excess chemical potential can be written as βμ_i_^ex^ = βμ_MSA_ + βμ_CS_ where the MSA contribution accounts for electrostatic correlations between the ions and is given by

21 $$\beta \mu_{MSA}=\frac{\kappa d\sqrt{1+2\kappa d}-\kappa d-{\left(\kappa d\right)}^{2}}{8\pi c_{t}d^{3}}$$

with *c*_t_ = *c*_a_ + *c*_s_, where *c*_a_ and *c*_s_ are the reservoir concentrations of acid and salt, respectively. The ionic diameter *d* is assumed, for simplicity, to be the same for all ionic species. In the present calculations we use *d* = 4 Å. The inverse Debye length is $\kappa =\sqrt{8\pi \lambda_{B}c_{t}}$ , where λ_B_ = *q* ^2^/ε_w_ *k*_B_ *T* is the Bjerrum length, which for water at room temperature is λ_B_ = 7.2 Å.

The Carnahan–Starling contribution accounts for the hard-sphere excluded-volume interactions and is given by

22 $$\beta \mu_{CS}=\frac{8\eta -9\eta^{2}+3\eta^{3}}{{\left(1-\eta \right)}^{3}}$$

For the monovalent electrolyte mixture considered here, the total ionic number density is 2*c*_t_ so that the ionic packing fraction entering the Carnahan–Starling expression is η = π*d* ^3^ *c*_t_/3.

This establishes the exact relationship between the reservoir activities and nominal electrolyte concentrations without requiring auxiliary bulk simulations.

### 3.2 Interfacial Charge Regulation and Reactive Monte Carlo Moves

When the confining boundaries are responsive, the fixed surface charge density is modulated by a local chemical equilibrium. We incorporate this responsive charge regulation by defining titratable surface groups (HA) located at the slab interfaces (*z* = ± *L*_w_/2). The protonation and deprotonation pathways are modeled via the standard dissociation reaction

23 $$\begin{array}{l} HA⇌H^{+}+A^{-}\\ K_{a}=\frac{a_{A^{-}}a_{H^{+}}}{a_{HA}} \end{array}$$

where A^–^ denotes a deprotonated surface site, and *K*_a_ is the intrinsic acid dissociation constant, which mathematically maps to the inverse of the internal partition function of the isolated HA state.

The total chemical free-energy change associated with pulling a hydronium ion out of the bulk reservoir and reacting it with an isolated, unprotonated surface group A^–^ is expressed as

24 $$\beta \Delta F_{p}=\ln\left(\frac{K_{a}}{c^{⊖}}\right)-\mu_{H^{+}}$$

where βμ_H^+^_ = ln­(*c*_H^+^_/*c* ^⊖^) + βμ_ex_ represents the total chemical potential of the hydronium ion in the reservoir, composed of its ideal concentration and excess nonideal parts. For the reverse deprotonation move, the chemical free-energy change carries the opposite sign, Δ*F*_d_ = −Δ*F*_p_.

The reactive protonation and deprotonation moves are attempted by randomly selecting a titratable site, flipping its ionization state, and accepting the transition with a probability equal to min­(1, ϕ_p_) or min­(1, ϕ_d_), respectively^70,71^

25 $$\begin{array}{l} \phi_{p}=\exp\left[-\beta \left(\Delta E_{ele}+\Delta F_{p}+q\varphi_{D}\right)\right]=10^{pK_{a}-pH}\;\exp\left[-\beta \left(\Delta E_{ele}+q\varphi_{D}\right)\right]\\ \phi_{d}=\exp\left[-\beta \left(\Delta E_{ele}+\Delta F_{d}-q\varphi_{D}\right)\right]=10^{pH-pK_{a}}\;\exp\left[-\beta \left(\Delta E_{ele}-q\varphi_{D}\right)\right] \end{array}$$

Here, p*K*_a_ = – log_10_(*K*_a_/*c* ^⊖^), and *q* represents the absolute elementary charge unit exchanged during the reaction. The explicit addition of the qφ_D_ work term ensures that the local proton affinity is coupled self-consistently to the macroscopic potential drop established between the slab and the reservoir.

### 3.3 Self-Consistent Potential Balancing and Sampling Protocol

Because the open ensemble allows both particle numbers and surface ionization states to fluctuate, the instantaneous total net charge *Q*_t_ of the simulation cell will deviate from zero during individual trial moves. To maintain physical consistency with a macroscopic system that is globally neutral on average, the simulation is initialized with a guess for the Donnan potential, φ_D_. Following each Monte Carlo step, this potential parameter is actively adjusted using a feedback algorithm to drive the system toward global neutrality

26 $$\varphi_{D}^{new}≔\varphi_{D}^{old}+\alpha Q_{t}$$

where α > 0 is a small, optimized tuning parameter that controls the numerical convergence rate and is typically chosen between 0.2 and 0.4 in this work. This iterative correction is maintained dynamically until the time-averaged total charge converges to zero within a tight prescribed tolerance (⟨*Q*_t_⟩ ≈ 0).

In practice, the system rapidly relaxes into a steady-state regime where *Q*_t_ exhibits only minor, symmetric thermal fluctuations around zero. To ensure robust thermodynamic equilibration, we simultaneously track both *Q*_t_ and the global energy *E*, assuming true equilibrium once the running average ⟨*E*⟩ becomes completely stationary. For the systems studied here, a window of 5 × 10^6^ individual particle and reactive moves is utilized exclusively for equilibration.

Following this equilibration phase, production sampling is initiated to extract structural and thermodynamic observables, including the effective net surface charge, the steady-state Donnan potential, and the highly resolved 1D ionic density profiles ρ_*i*_(*z*). To minimize statistical correlation between consecutive configurations, data samples are recorded sparingly – every 3000 complete Monte Carlo sweeps. A total production pool of 20 000 statistically independent, uncorrelated snapshots is then accumulated to calculate final ensemble averages.

## 4 Simulation Model and Numerical Validation

### 4.1 Physical System and Simulation Parameters

To validate the regularized Ewald electrostatics framework, we perform Monte Carlo simulations of a model slit nanopore and compare the structural observables directly against exact numerical solutions of the nonlinear Poisson–Boltzmann (PB) equation, which is known to be very accurate for 1:1 electrolyte. The physical system consists of a highly charged planar plate positioned symmetrically at the center (*z* = 0) of a confined slab domain. The slab is bounded by rigid, impenetrable walls located at *z* = ± *L*_w_/2, which restrict the transverse motion of the mobile ions, see Figure 2. Periodic boundary conditions are applied exclusively along the lateral *x* and *y* dimensions for the physical system, while the region beyond the macroscopic interface at *z* = +*L*_w_/2 transitions to an external implicit bulk electrolyte reservoir. However, to leverage the computational efficiency of 3D periodic electrostatics within our framework, an artificial periodic replication is also introduced along the confinement axis characterized by a supercell width *L*_*z*_. In our numerical simulations, we explicitly set the lateral dimensions to be equal (*L*_*x*_ = *L*_*y*_) and enforce *L*_*z*_ = 2*L*_*x*_. This configuration maintains an implicit, wide vacuum region between the physical pore boundaries (±*L*_w_/2) and the artificial boundaries of the supercell (*L*_*z*_), ensuring that the regularized fields decouple cleanly and the unphysical replica artifacts vanish. The general structural layout of this open simulation cell is illustrated schematically in Figure 2.

**Figure 2:**
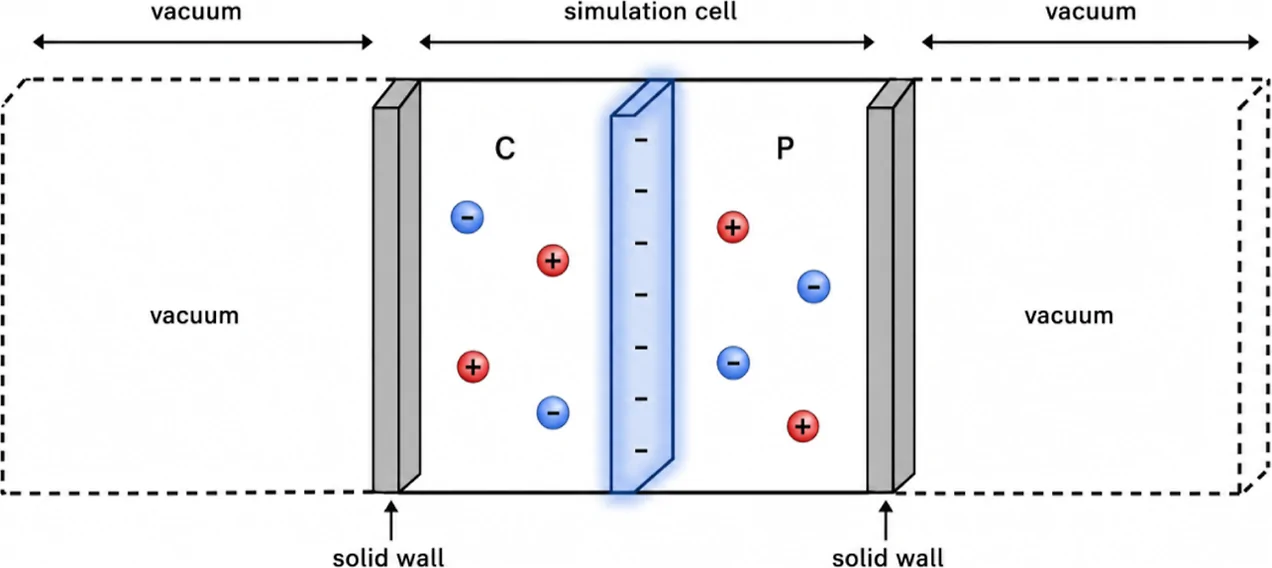
Schematic representation of the open slab simulation cell. Periodic boundary conditions are applied along the lateral *x* and *y* axes (*L*_*x*_ = *L*_*y*_ = 100 Å), while the righthand wall (*z* = +*L*_w_/2) couples seamlessly with an external reservoir.

The central plate is modeled with a uniform surface charge density of σ_q_ = 230 mC m^–2^. In practice, we use *N*_s_ fixed point charges located at *z* = 0 and arranged on a simple two-dimensional square lattice in the *xy* plane. The number of surface sites is chosen to reproduce the prescribed average surface charge density according to σ_q_ = *N*_s_ *q*_s_/(*L*_*x*_ *L*_*y*_), where *q*_s_ is the charge of each surface site.

The implicit external bulk reservoir contains a symmetric 1:1 electrolyte at a fixed salt concentration of *c*_bulk_ = 50 mM. The lateral dimensions of the simulation cell are held constant at *L*_*x*_ = *L*_*y*_ = 100 Å, while the pore width is systematically varied (*L*_w_ = 100, 80, 50, and 20 Å) to probe different regimes of nanoscale spatial confinement.

The solvent environment is treated as a uniform continuum medium characterized by a bulk dielectric constant of ϵ_w_ = 78, which corresponds to a standard Bjerrum length 7.2 Å at room temperature. The electrolyte is simulated using the classical primitive model, where the mobile ions are represented as hard spheres of valence *z*_*i*_ = ± 1 with a fixed ionic radius of *R*_ion_ = 2 Å. For each pore configuration, the simulation is equilibrated for a window of 5 × 10^6^ complete Monte Carlo steps, followed by a production phase of 20 000 statistically independent sampling blocks to accumulate highly resolved ensemble averages.

### 4.2 Ionic Density Profiles and Donnan Potentials

We first examine the spatial distribution of the mobile ions within the slit pore. Figure 3 displays a direct comparison between the discrete counterion and co-ion density profiles ρ_*i*_(*z*) extracted from the regularized simulations and the corresponding mean-field profiles calculated via the nonlinear Poisson–Boltzmann equation under identical boundary constraints.

**Figure 3:**
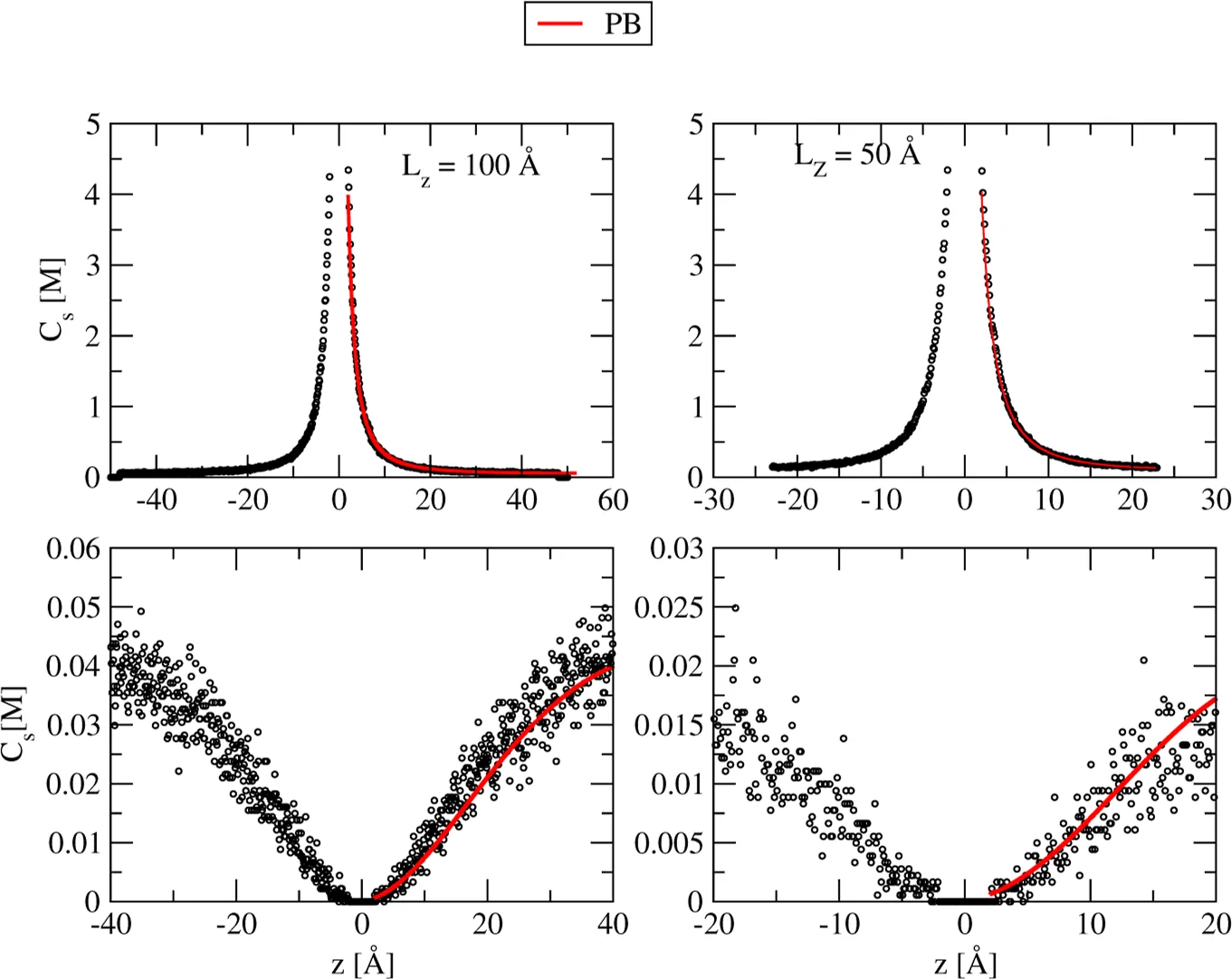
Comparison of the local ionic density profiles ρ_*i*_(*z*) between GCMC simulations (symbols) and numerical solutions of the nonlinear Poisson–Boltzmann equation (solid lines) for two distinct pore widths (*L*_w_ = 100 Å and *L*_w_ = 50 Å) and reservoir salt concentration of 50 mM. Top panels shows counterion profiles and bottom co-ion.

As demonstrated clearly in Figure 3, the agreement between the regularized 3D Ewald simulation profiles and the PB results is excellent across the entire width of the pore. The regularized Green’s function successfully captures the intense screening layer near the charged central plate without introducing *z*-periodic distortions, confirming that the structural mapping onto the 3D periodic architecture handles the local electrostatics correctly.

We next evaluate the equilibrium Donnan potential φ_D_ as a function of the pore width *L*_w_. The Donnan potentials established during the simulation runs are compared directly with the values predicted by integrating the Poisson–Boltzmann equation. These numerical benchmarks are summarized in Table 1.

**Table 1:** Comparison of the Equilibrium Donnan Potential φ_D_ (in Units of *k*_B_ *T*/e) Obtained from Our Regularized Simulation Framework and the Nonlinear Poisson–Boltzmann Equation Across Varying Pore Widths *L*_w_

*L*_w_ (Å)	regularized simulation	Poisson–Boltzmann
100	–0.16043	–0.1585
80	–0.3453	–0.3273
50	–0.9474	–0.9206
20	–2.30	–2.386

Calculating the correct Donnan potential requires an electrostatics formulation free of cell-size artifacts, highlighting the necessity of our regularization scheme when compared to standard, uncorrected 3D periodic boundary conditions.^75^ If one attempts to utilize an uncorrected 3D Ewald summation with a standard vacuum gap along the *z*-axis for non-neutral cell states (*Q*_net_ ≠ 0), the simulation fails to reach a stable equilibrium. This unphysical behavior arises because standard 3D Ewald routines automatically inject a uniform neutralizing background cloud across the entire volume *V* = *L*_*x*_ *L*_*y*_ *L*_*z*_.^68^ When integrated along the confinement axis, this symmetric background charge distribution generates a spurious parabolic electrostatic potential artifact across the simulation domain. This unphysical potential distortion modifies the electrostatic work associated with single-ion insertion moves, weakening the natural electrostatic potential barrier at the pore boundary (*z* = *L*_w_/2) that physically penalizes excessive charge accumulation. Without the correction terms developed in this work to match the absolute electrostatic potential to zero in the bulk reservoir region (*z* ≥ *L*_w_/2), the system loses the essential electrostatic feedback required to restrict incoming species. Consequently, an unconstrained charge accumulation occurs where mobile ions continuously enter the cell without limit, preventing the simulation from reaching a physical thermodynamic equilibrium.

Our regularized formulation eliminates this cell-size parabolic artifact and restores numerical convergence. By explicitly subtracting the parabolic potential contributions of the 3D background cloud and incorporating the geometric Bethe potential alongside exact reservoir boundary conditions, the unphysical vacuum terms decouple completely. The system systematically converges onto the true 2D periodic Green’s function, ensuring that the calculated Donnan potential and structural properties reflect genuine thermodynamic equilibrium with the reservoir rather than arbitrary simulation cell dimensions (*L*_*z*_). As a result of this stable physical foundation, our regularized simulation framework successfully achieves a steady state and demonstrates close agreement with accurate theoretical predictions of the nonlinear Poisson–Boltzmann equation for 1:1 electrolytes across all confinement scales, as summarized in Table 1.

## 5 Interfacial Charge Regulation and Surface Titration

### 5.1 Charge Regulation Model

To demonstrate the power of our reservoir-matched Ewald framework in handling complex, chemically responsive boundaries, we investigate the phenomenon of charge regulation inside a slit nanopore featuring an asymmetric surface functionalization. We consider a single, structurally fixed planar surface located at the left pore boundary, *z* = −*L*_w_/2, while the opposing boundary at *z* = +*L*_w_/2 acts as a hard neutral wall defining the geometric limit of the slit. Rather than possessing a static, predetermined surface charge density, the surface is functionalized with a discrete number *Z* of titratable weak acidic groups (AH) of p*K*_a_. The system remains coupled to an implicit external reservoir through the interface at the right boundary, *z* = +*L*_w_/2, allowing for the dynamic exchange of mobile counterions, co-ions, and hydronium ions.

The local ionization state of each individual surface site on the plate is governed by the responsive chemical equilibrium

27 $$AH+H_{2}O⇌A^{-}+H_{3}O^{+}$$

As the local environment changes, the surface groups dynamically adjust their charge state between neutral (AH) and negatively charged (A^–1^) forms. Consequently, the net effective surface charge of the plate is not a fixed boundary condition but a fluctuating thermodynamic observable that depends self-consistently on the local electrostatic potential, the concentration of protons inside the pore, and the bulk reservoir parameters (pH and salt concentration).

### 5.2 Reactive Acceptance Criteria under Confinement

The dynamic ionization of the *Z* surface sites is simulated using reactive Monte Carlo moves, as detailed in Section 3. During a protonation or deprotonation trial, a titratable site on the plate (*z* = −*L*_w_/2) is selected at random. Because our regularized Green’s function $G_{3dr}$ explicitly references the absolute potential to the zero-potential bulk reservoir, the work associated with transferring a hydronium ion between the reservoir and the surface site at coordinate *z* = −*L*_w_/2 is rigorously captured by the work term ±*q*φ_D_, where *q* is the elementary charge unit.

The acceptance parameters ϕ_p_ (protonation) and ϕ_*d*_ (deprotonation) switch the charge of a site by Δ*q* = ± 1 according to eq 25. Crucially, the deprotonation of a site on the plate increases the local negative potential inside the pore, which generates a significant electrostatic energy penalty Δ*E*_ele_ for subsequent deprotonation events. This regularized feedback loop ensures that ion–ion correlations and discrete surface-charge repulsions are captured beyond the limitations of standard mean-field Poisson–Boltzmann theory.

### 5.3 Observables: Effective Valence and Differential Profiles

The primary thermodynamic observable used to characterize the responsive nature of the pore is the mean effective surface valence of the plate, *Z*_eff_, defined as the ensemble average of the number of deprotonated, active sites

28 $$Z_{eff}=\left\langle \sum_{k=1}^{Z}\delta_{\alpha_{k},A^{-}}\right\rangle$$

where $\delta_{\alpha_{k},A^{-}}=1$ if site *k* is ionized and 0 if it is neutral. The structural properties of the confined fluid are systematically tracked via 1D ionic density profiles ρ_*i*_(*z*) for all mobile electrolyte species and hydronium ions across the pore domain −*L*_w_/2 ≤ *z* ≤ *L*_w_/2.

The regularized single-ion grand canonical framework developed here is well suited to handle such responsive interfaces. In traditional paired-ion GCMC schemes, sampling a local ionization step requires the simultaneous insertion or deletion of a neutral ion pair to enforce strict cell neutrality, which leads to low acceptance probabilities near dense electric double layers. By allowing the net cell charge *Q*_t_ to fluctuate during trial operations, our method permits the ionization states of the functional groups and the local counterion screening cloud to adjust self-consistently and independently, providing an efficient route to evaluate nonideal electrostatic correlations that modify the effective electromotive force (emf) of the responsive channel.

Comparing *Z*_eff_ and the local density profiles against the theoretical mean-field framework developed in our previous work^76–78^ provides validation of the methodology. As shown in Figures 4 and 5, the agreement between the regularized grand canonical reactive simulations and our theoretical results is excellent. Our model captures the shape of the titration curve under confinement as well as the structure of the counterion screening layer adjacent to the responsive plate. Knowledge of the Donnan potential also permits us to calculate the pH inside the slab, which differs from that of the reservoir because of the Donnan potential difference between the two

29 $${pH}_{slab}={pH}_{res}+\frac{e\varphi_{D}}{k_{B}T\;\ln10}$$

**Figure 4:**
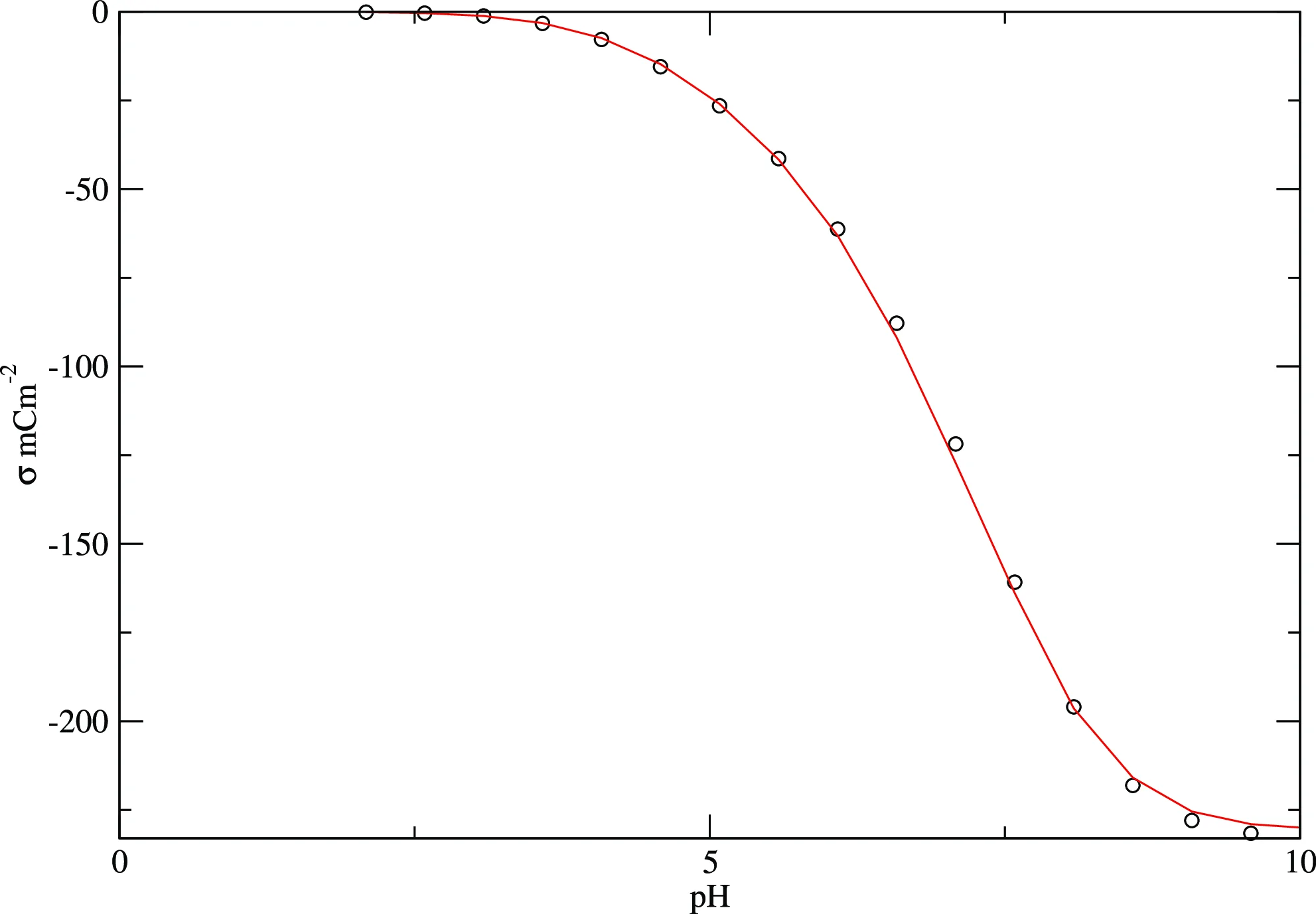
Titration curve, as a function of pH in reservoir, for a surface located at *z* = −*L*_w_/2 with *L*_w_ = 100 Å and a reservoir salt concentration of 50 mM. The total nominal surface density of titratable sites corresponds to 230 mC m^–2^, *Z* = 144. Solid lines represent predictions of the theoretical framework developed in our previous work,^76–78^ demonstrating excellent agreement with the simulation data (symbols). In all cases *L*_*x*_ = *L*_*y*_ = 100 Å, *L*_*z*_ = 2*L*_*x*_, and p*K*_a_ = 5.4.

**Figure 5:**
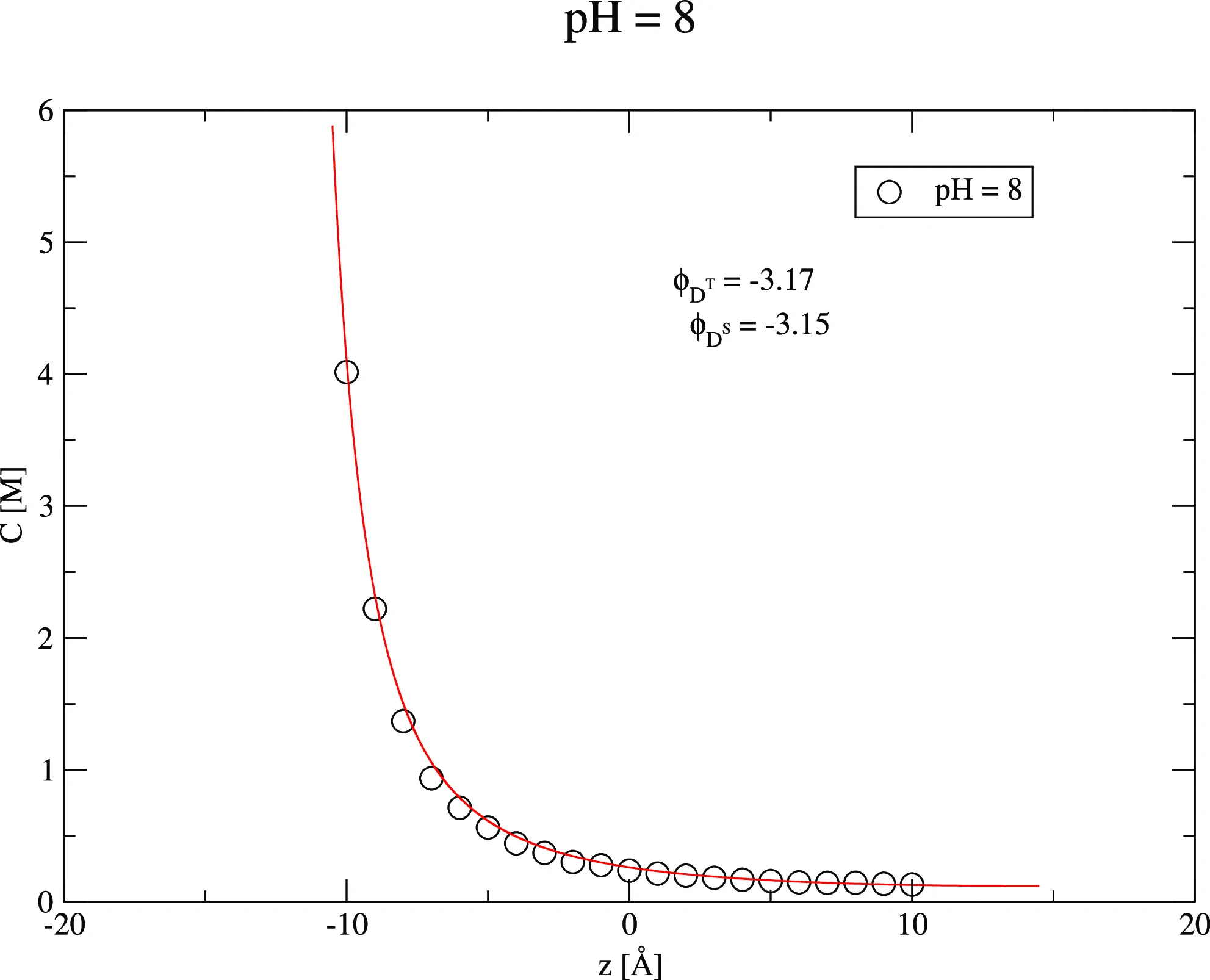
Counterion density profile ρ_*i*_(*z*) for a narrow pore of width *L*_w_ = 25 Å and surface located at *z* = −*L*_w_/2, in contact with a reservoir of salt concentration of 5 mM. The solid line indicates the analytical profile derived via our previous theoretical framework,^76–78^ symbols are the simulation points. The theoretical value of the Donnan potential was found to be βqφ_D_ = −3.17, while the simulation yields βqφ_D_ = −3.15. The parameters are *L*_*x*_ = *L*_*y*_ = 100 Å and p*K*_a_ = 5.4 and *L*_*z*_ = 2*L*_*x*_.

While mean-field approximations are accurate for the monovalent electrolyte conditions examined here, our simulation framework provides the machinery to capture strong ion–ion correlations, fluctuating polarization fields, and localized packing constraints, where mean-field charge regulation models break down.

## 6 Conclusions

We have developed a computationally efficient electrostatic framework designed specifically for simulating open, non-neutral confined fluid interfaces in thermodynamic equilibrium with an external electrolyte reservoir. Rather than relying on computationally demanding 2D Lekner or explicit 2D Ewald summation methods, our approach modifies the standard 3D periodic Ewald architecture. By isolating the unphysical 3D periodic background artifacts, incorporating the geometric Bethe potential, and applying asymmetric reservoir-matching boundary conditions at the open interface (*z* = +*L*_w_/2), we derived a regularized Green’s function and a total global energy functional that remain valid for globally non-neutral configurations possessing an arbitrary net system charge (*Q*_t_ ≠ 0).

The accuracy and physical validity of the framework were established through a series of numerical benchmarks. First, evaluating the self-potential limit against a one-component 2D plasma square lattice confirmed that our regularized 3D formulation systematically converges to the exact analytic 2D Madelung limit as the supercell width (*L*_*z*_) increases relative to the lateral dimensions. Second, grand canonical Monte Carlo simulations of a model slit nanopore demonstrated close structural agreement with exact numerical solutions of the nonlinear Poisson–Boltzmann equation and our previously developed analytical theory for charge-regulated monovalent electrolyte systems.^76–78^ While uncorrected 3D Ewald slab simulations introduce spurious parabolic potential artifacts that alter boundary work terms, scale with the vacuum domain, and lead to unconstrained particle accumulation during open-ensemble sampling, our regularized scheme completely eliminates this cell-width dependence. The vacuum interactions decouple cleanly, yielding invariant ionic density profiles and reproducible macroscopic Donnan potentials across all confinement scales.

Crucially, because no existing alternative Ewald formulation can directly determine the electrostatic potential drop of an open, non-neutral slab configuration, treating the Donnan potential as an explicit operational parameter offers a direct method to quantify nonideal electrostatic correlation and ionic packing corrections to the microscopic electromotive force (emf) of confined channels. Furthermore, by decoupling the grand canonical transition matrix into independent, single-ion trial moves, this methodology circumvents the sampling bottlenecks and screening distortions associated with traditional neutral-pair insertion schemes. As demonstrated in Section 4, this regularized formulation is particularly well suited for reactive Monte Carlo simulations of chemically responsive boundaries, such as weak acid functionalized pore walls. While validating mean-field predictions under the monovalent conditions studied here, this discrete structural framework establishes the necessary computational machinery to explore regimes where mean-field approximations fail, including systems with multivalent counterions or elevated surface charge densities. Finally, due to its structural generality and explicit reference of potentials relative to an external bulk phase, this framework can be directly extended to molecular simulations with explicit solvent models in slab geometries.
